# Characterizing the consensus residue specificity and surface of BCL-2 binding to BH3 ligands using the Knob-Socket model

**DOI:** 10.1371/journal.pone.0281463

**Published:** 2023-02-16

**Authors:** Jennifer Yi, Vivian Kellner, Hyun Joo, Nathaniel Chien, Shivarni Patel, Zaina Chaban, Jerry Tsai

**Affiliations:** 1 Department of Molecular and Cell Biology, UC Berkeley, Berkeley, California, United States of America; 2 Department of Chemistry, UC Davis, Davis, California, United States of America; 3 Department of Chemistry, University of the Pacific, Stockton, California, United States of America; 4 Computer Science Department, Stanford University, Stanford, California, United States of America; Italian National Research Council, ITALY

## Abstract

Cancer cells bypass cell death by changing the expression of the BCL-2 family of proteins, which are apoptotic pathway regulators. Upregulation of pro-survival BCL-2 proteins or downregulation of cell death effectors BAX and BAK interferes with the initiation of the intrinsic apoptotic pathway. In normal cells, apoptosis can occur through pro-apoptotic BH3-only proteins interacting and inhibiting pro-survival BCL-2 proteins. When cancer cells over-express pro-survival BCL-2 proteins, a potential remedy is the sequestration of these pro-survival proteins through a class of anti-cancer drugs called BH3 mimetics that bind in the hydrophobic groove of pro-survival BCL-2 proteins. To improve the design of these BH3 mimetics, the packing interface between BH3 domain ligands and pro-survival BCL-2 proteins was analyzed using the Knob-Socket model to identify the amino acid residues responsible for interaction affinity and specificity. A Knob-Socket analysis organizes all the residues in a binding interface into simple 4 residue units: 3-residue sockets defining surfaces on a protein that pack a 4^th^ residue knob from the other protein. In this way, the position and composition of the knobs packing into sockets across the BH3/BCL-2 interface can be classified. A Knob-Socket analysis of 19 BCL-2 protein and BH3 helix co-crystals reveal multiple conserved binding patterns across protein paralogs. Conserved knob residues such as a Gly, Leu, Ala and Glu most likely define binding specificity in the BH3/BCL-2 interface, whereas other residues such as Asp, Asn, and Val are important for forming surface sockets that bind these knobs. These findings can be used to inform the design of BH3 mimetics that are specific to pro-survival BCL-2 proteins for cancer therapeutics.

## Introduction

**Fig 1:**
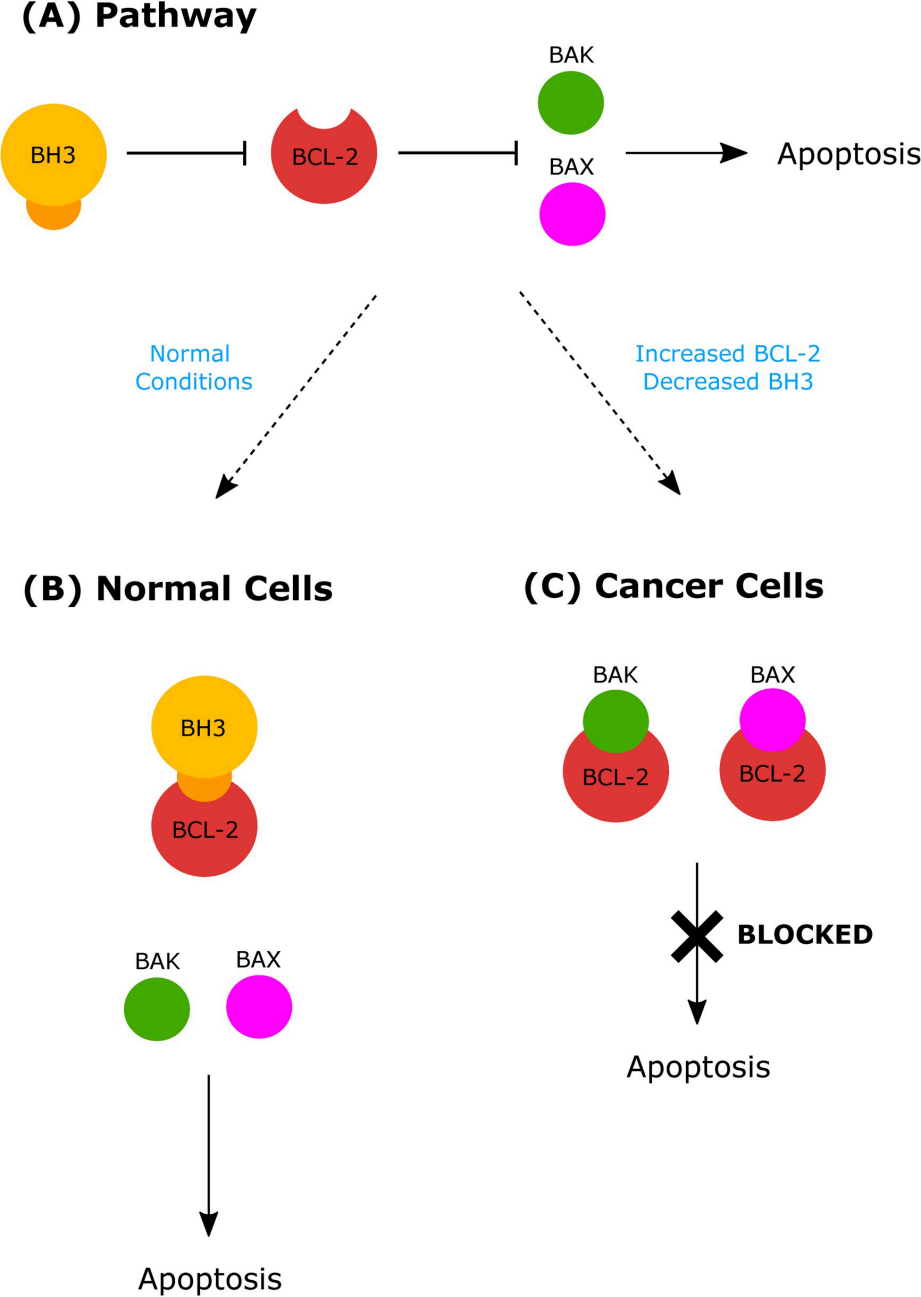
BCL-2-regulated apoptosis. (A) The BCL-2-regulated apoptotic pathway involves the following proteins pro-survival BCL-2 proteins, pro-apoptotic BH3-only proteins, and apoptosis effectors BAX/BAK proteins. Lines indicate interactions between molecules. (B) In healthy cells, the pro-survival BCL-2 proteins sequester BAX and BAK proteins so that these effectors cannot initiate apoptosis. Normally, apoptosis occurs indirectly due to the pro-survival BCL-2 proteins being bound by BH3-only proteins, which prevents the pro-survival BCL-2 proteins from binding and inhibiting BAX/BAK. Subsequently, the free BAX/BAK can initiate apoptosis. (C) In cancer cells, the loss of BH3-only proteins or the increase in pro-survival BCL-2 proteins causes BAX/BAK to be directly bound by pro-survival BCL-2 proteins, preventing the initiation of apoptosis.

One of the many hallmarks of cancer is the evasion of cell death. While this process can occur through many pathways, interruption the initiation of mitochondrial apoptosis through the altered expression of the BCL-2 protein family of proteins is found in many cancers [1]. This family of proteins is classified into 3 groups based on their function in mitochondrial apoptosis (Fig 1): pro-survival BCL-2 proteins, pro-apoptotic BH3-only proteins, and the apoptosis effector proteins BAX and BAK [1]. The pro-survival BCL-2 was initially discovered for its role in B-cell lymphoma, hence its name B-cell lymphoma 2, and it was shown to contribute to tumor initiation and progression. In the human genome, different homologs of the pro-survival BCL-2 protein have been identified: BCL-2, BCL-x_L_, MCL-1, BCL-w, BFL-1/A1, BCL-B; in addition to a number of related proteins that share significant amino acid sequence similarity [2]. As diagrammed in Fig 1, the pro-survival BCL-2 proteins contain a hydrophobic groove that binds both pro-apoptotic BH3-only proteins as well as the effector proteins BAX and BAK, and competition for this hydrophobic binding site determines mitochondrial apoptosis. Normally, cells avoid apoptosis when the pro-survival BCL-2 protein sequesters BAX and BAK in the hydrophobic binding site from effecting their cell-death inducing, signal transduction cascade. BH3-only proteins refer to members of the BCL-2 protein family that contain only one of the four BCL-2 homology (BH) regions, the BH3 domain. Under cell stress, apoptosis occurs because BH3-only proteins bind the pro-survival BCL-2 proteins in their hydrophobic groove, thus freeing BAX and BAK proteins to initiate apoptosis [1]. Cancer cells inhibit the mitochondrial apoptosis pathway by upregulation of pro-survival BCL-2 proteins, which results in bound BAX and BAK proteins and inhibition of cellular apoptosis [1,3]. Because of its role in regulating apoptosis through binding, this hydrophobic groove on pro-survival BCL-2 proteins is an attractive target for anti-cancer drugs that can counteract the overexpression of the pro-survival BCL-2 proteins. Called BH3-mimetics, this class of drugs binds in the hydrophobic groove like BH3-only proteins and allows free BAX and BAK to cause programmed cell death. The goal of this research is to characterize the packing interface between pro-survival BCL-2 proteins and the BH3 α-helix using the Knob-Socket method [4–6] to inform the future development of cancer therapeutics. The Knob-Socket method to precisely map the α-helical protein ligand interface between BCL-2 and the BH3 domain for the specific amino acid residues that define complementary surfaces of affinity as well as specificity of packing. From this analysis, conserved interactions can be identified to inform the eventual design of effective inhibitors of the anti-apoptotic signal in cancer cells.

As mentioned above, overexpression of pro-survival BCL-2 homologs have been found in many cancers, and so develop of BH3 mimetics as anti-cancer drugs is an active field of study [7]. In particular, lymphomas and leukemias are regularly found to have upregulation one or more of the proteins BCL-2, MCL-1, and BCL-x_L_. In addition, BCL-2 and BCL- x_L_ overexpression contributes to cancer in many solid tumors of the brain, breast, lung, and glioma cancers. Increased BCL-w is found in many cancers of the digestions system, including gastric and colorectal cancers. Since the pro-survival BCL-2 proteins are involved in such a broad range of cancers and intervention with a BH3 mimetic would cause those cells to undergo programmed cell death, therapeutics targeting the pro-survival BCL-3’s hydrophobic groove with small molecule compounds as BH3 mimetics. The major problem is toxicity to normal cells, because the BH3 mimetics would target healthy cells as well. So, drug development has shifted towards BH3 mimetic specificity. As one of the first, navitoclax targets BCL-2, BCL- x_L_, and BCL-w, but lack of specificity killed platelet cells. Venetoclax was found to be more specific for BCL-2 and has shown success as part of a drug cocktail in combating leukemias. For BCL- x_L_, several compounds have been developed and some have shown success in killing certain cancer cells. These are still in the early stages of the drug development process. The BH3 mimetics targeting MCL-1 have shown promise, but the cross toxicity with heart cells has halted the progression of these drugs. The common theme for all of these has been large scale screening of compounds instead of directed identification of ligands that would bind the pro-survival BCL-2’s hydrophobic groove.

BH3 mimetics are a type of anti-cancer drug that mimic the binding of BH3 domain protein to the hydrophobic groove of pro-survival BCL-2 proteins. BH3-only proteins and their distant relatives all share the BH3 domain, which adopts an α-helical fold when bound to the pro-survival BCL-2 protein [8,9]. The BH3-only domain acts as an α-helical ligand to the hydrophobic groove formed by several α-helices of the pro-survival BCL-2 protein [10]. Specifically, the interaction between the pro-apoptotic BH3-only protein and the pro-survival BCL-2 protein consists of the hydrophobic face of an amphipathic α-helix of the BH3 domain inserting into the aforementioned hydrophobic groove of the pro-survival BCL-2 protein [11]. These BH3-only proteins display a diverse array of binding specificities to the pro-survival BCL-2 proteins [11]. While all BH3-only proteins can indirectly activate BAX/BAK through the inhibition of pro-survival BCL-2 proteins, some of these BH3-only proteins can activate BAX/BAK through direct binding [3,8]. ‘Activator’ BH3-only proteins refer to those proteins that directly bind and activate BAX/BAK, while ‘sensitizer’ BH3-only proteins bind the pro-survival proteins and liberate BAX/BAK [9]. Therefore, it appears that both the indirect and direct activation of BAX/BAK are critical for inducing apoptosis. A BH3 mimetic would promote cancer cell apoptosis by binding pro-survival proteins and allowing for the displacement of activator BH3-only proteins, which could then directly activate BAX/BAK as well as the saturation of pro-survival proteins that would otherwise inhibit BAX/BAK. The basis of the BH3 mimetic relies on disrupting the interaction of the pro-apoptotic BH3 domain with the hydrophobic pocket of the pro-survival BCL-2 proteins, thus permitting BAX or BAK to initiate programmed cell-death.

Many previous studies of the binding interaction between pro-survival and pro-apoptotic proteins screened a library of peptides for sequences that are specific to a certain BCL-2 protein, and then optimized the most promising sequences in some capacity to improve upon their binding affinities and specificities to said BCL-2 protein. Lee *et al*. [12] examined the binding determinants of the BCL-2 protein and BH3 domain interaction by screening the pro-survival BCL-2 protein Mcl-1 against randomized phage-displayed peptide libraries to identify novel peptides. After initial peptide sequences selective for Mcl-1 were identified, binding to pro-survival BCL-2 targets was improved through sequence alterations and extensions. Another study by Foight *et al*. modified Mcl-1-specific peptides selected from a yeast-surface display library by replacing a glycine residue in the sequence with a threonine. Of the resulting peptides, the most specific one was able to bind Mcl-1 with 40-fold or greater specificity compared to four other human BCL-2 paralogs [13]. Stewart *et al*. screened a library of Stabilized α-Helix of BCL-2 domains (SAHBs) and identified the Mcl-1 BH3 helix as a potent inhibitor of Mcl-1. The affinity of this helix to Mcl-1 was further optimized through the application of hydrocarbon stapling [14]. Other strategies that do not utilize the traditional experimental screening method in the design of BH3 mimetics have also been explored. Pinto *et al*. used a pharmacophore-based strategy to rationally pinpoint inhibitors of the pro-survival BCL-xL protein. By studying the interaction between BCL-xL and the BH3 domains of different pro-apoptotic proteins, four pharmacophoric points that contribute to the recognition between BCL-xL and the BH3 domain were identified. These features were defined as two hydrophobic groups and two hydrogen bond acceptors on the BH3 ligand. After screening multiple 3D databases for the pharmacophoric features, two compounds that were able to disrupt the inhibitory nature of BCL-xL were identified [15]. Friberg *et al*. employed NMR-based screening of a fragment library to identify two classes of chemically distinct small molecules that bind to different sites on Mcl-1. Members of these two classes were linked together to create lead compounds that selectively bind Mcl-1 with a dissociation constant of <100 nM [16].

In this study, the Knob-Socket model was used to map the protein-ligand interactions between pro-survival BCL-2 protein paralogs and the BH3 domains of BH3-only protein paralogs to inform the design of a peptide that can mimic the BH3 domain and bind to the pro-survival BCL-2 protein. This method has been used before to successfully identify the amino acid determinants in the successful design of peptides that mimic antibody binding [5,6]. These instances demonstrate the use of the Knob-Socket model to rationally design peptides that specifically bind their intended targets.

**Fig 2:**
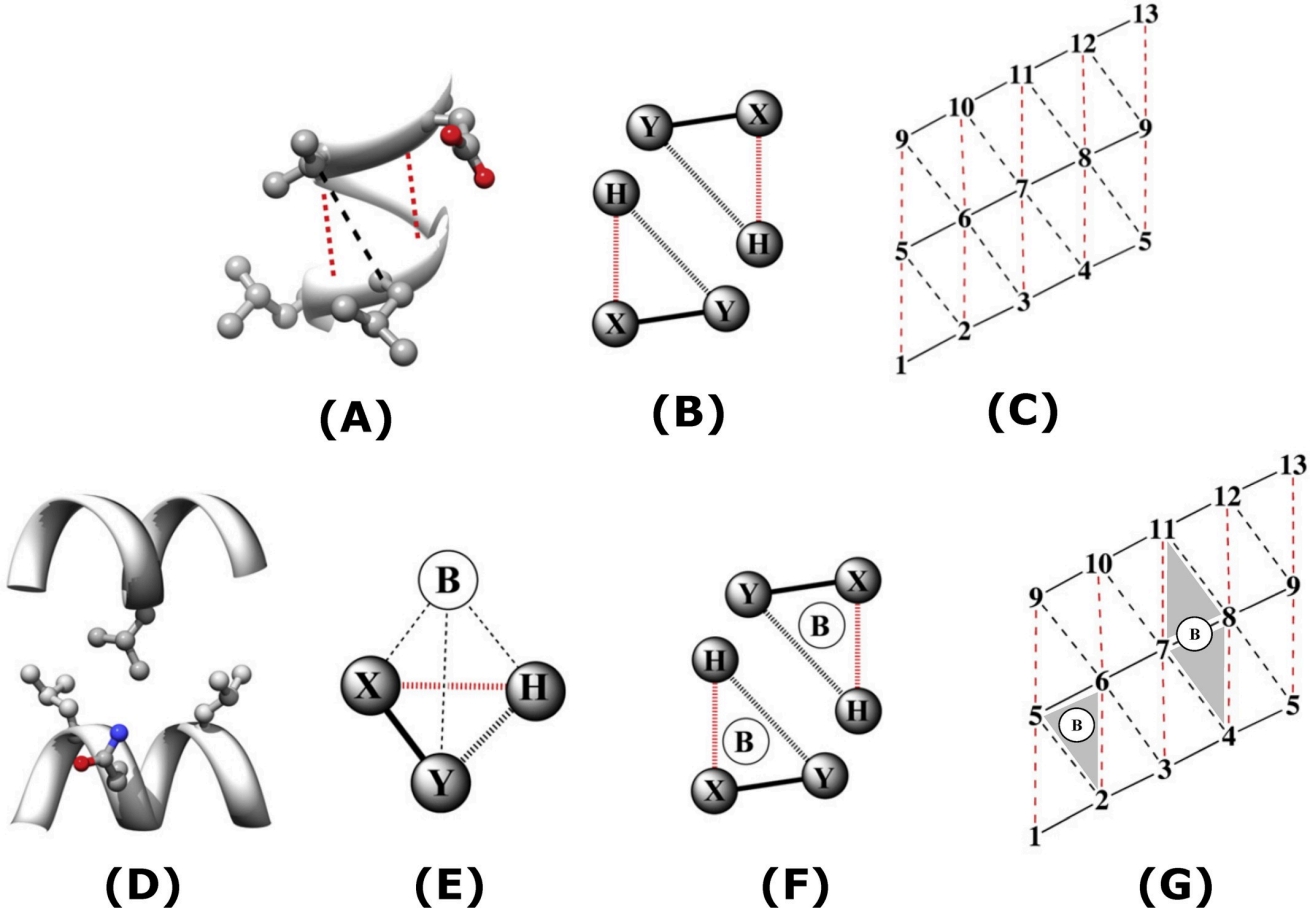
Visual representations of the Knob-Socket model. (A) 3-dimensional model of a socket shown on an α-helix. (B) 2-dimensional representation of a socket formed by the residues X, Y, and H. The 3 residues clearly form a surface that can pack another residue. (C) α-helical lattice showing the sockets formed on an α-helix surface, with numbers representing socket residues. (D) 3-dimensional model of a knob on one helix (upper) packing into a socket on another helix (lower). (E) Geometric model showing the tetrahedral arrangement of the knob-socket complex. (F) 2-dimensional representation of the knob-socket motif with three socket residues X, Y, and H packed against a knob residue B from another helix. (G) 2-dimensional lattice representation of a socket (formed by residues 2, 5, and 6) filled by knob residue B, and a pocket (formed by residues 4, 7, 8, and 11) filled by knob residue B. When a knob residue B packs into 2 contiguous sockets, the overall surface formed by those residues is denoted a pocket.

The Knob-Socket model characterizes protein packing at the tertiary and quaternary level by organizing interactions into repetitive patterns of single amino acid “knobs” packing into multiple amino acid “sockets” (Fig 2). In this system specifically, the knob-socket motif refers to a four-residue tetrahedral motif, where a knob residue on one α-helix packs into a three-residue socket on another α-helix [4]. The three-residue sockets essentially act as a surface target for a single residue knob to pack. The repetitive patterns of α-helix sockets provides a uniform packing lattice made by the amino acid residues [4]. By analyzing the points of contact between helices in an interaction, it is possible to determine patterns of packing specificity. While many computational studies that aim to optimize the design of BH3 mimetics have been performed, this study uniquely describes a model of residue and packing specificity between the hydrophobic pocket of the pro-survival BCL-2 protein and the BH3 domain helices.

The interplay between pro-apoptotic and pro-survival proteins is a complex one, as the diversity of BCL-2 family proteins can vary across tissues and cell types. However, the interface is a single α-helix of the BH3 domain packing into a hydrophobic groove formed by the α-helices of a BCL-2 protein. Using a Knob-Socket (KS) analysis of the packing interface between pro-survival BCL-2 proteins and BH3 domains, the goal of this research is to define the exact residues responsible for protein interaction affinity and specificity. In particular, the Gly residues in both the ligand and binding groove play an important role in binding orientation specificity.

## Methods

### Knob-socket analysis

Based on the Knob-Socket analysis of α-helical packing [4], the knobs and sockets involved in each BCL-2:BH3 interaction were defined. The crystal structures of all studied interactions [4] were visualized using the UCSF Chimera program package [17]. Detailed in previous papers [4,18–20], the Knob-Socket analysis begins by precisely defining residue cliques: a set of residues that all contact each other. Atomic contacts were calculated from Voronoi polyhedra, and residue-residue contacts were built up from the atomic contact information. The resulting Delaunay tessellation of residue interactions defines a contact graph between residues, where cliques can be defined. These relative packing cliques precisely define a set of residues that all contact each other and classify them based on contact order. The cliques are classified into the three-residue sockets and four-residue ‘knobs packing into a socket’. For α-helices, these 2:1 cliques consist of main-chain interactions of “2” neighboring residues X and Y connected by a covalent peptide bond, where the X residue shares the “:1”, helical *i* to *i* + 4 hydrogen bond with residue H. Residues H and Y share only side-chain packing interaction between them and are separated by three residues in the sequence. With a few exceptions, these α-helical cliques exhibit the same connectivity in two orientations, the XY:H socket and the H:YX socket, where the first residue listed indicates the residue that is lowest in sequence position and the “:” indicates that residue H is hydrogen bonded. The bonding interactions of the two socket orientations are incorporated to create the lattice representation of intra-helical packing of the two XY:H and H:YX sockets. In addition to the covalent peptide bonds and hydrogen bonding, the packing between the *i* and *i* + 3 residues is also shown, as it contributes to the regularity of the socket pattern. For inter-helical packing, the four-residue cliques 2:1 + 1 builds off the three-residue 2:1 socket on one α-helix together with a +1 knob B residue from another α-helix. Following the knob-socket model, the knob B on one α-helix packs into the three residue XY:H or H:YX socket presented by another α-helix to form a tetrahedral configuration. These knob-socket motifs are represented on the lattice by placing the knobs into the center of the appropriate sockets. These designations are relative, as residues can participate in more than one role in different knob-sockets. Following the process outlined above, the resulting packing topology maps that visually represent the knob-socket interactions between BH3 helices and BCL-2 proteins were created individually by hand using postscript editing software Adobe Illustrator and Inkscape. The Knob-Socket analysis provides a simple mapping of the residues involved in the binding interfaces of the BCL-2:BH3 interactions. All BCL-2:BH3 protein complexes that were mapped are listed in Table 1 with their corresponding PDB IDs. All 19 maps are provided in the supplementary material. From these 19 maps, more detailed analysis was performed and a model of BCL-2:BH3 binding was developed.

**Table 1:** List of interactions between various pro-survival BCL-2 proteins and the different BH3 ligands examined in this study.

Studied BCL-2:BH3 Interactions		
BCL-2 Protein	BH3 Peptide	PDB ID
Mcl-1	Bim	2pqk
	Bax	3pk1
	Bid	2kbw
	Bim I2dY	3kj0
	Bim F4aE	3kj2
BFL-1 / BCL-2 A1	Noxa	3mqp
	Bid	2voi
	Bim	2vm6
	Bak	2voh
Murine BFL-1	BMF	2vog
	Puma	2vof
BHRF1	Bim	2wh6
	Bak	2xpx
BCL-xL	Bim	3fdl
	Bak	1bxl
	Bad	1g5j
	Bim L12F	3io8
	Beclin-1	2pon
BCL-2	Bax	2xa0

### Distance calculations

Distances between sockets were calculated between the centroids of the sockets. Centroid of a socket is defined by the average of the Cβ coordinates of residues in that socket.

### Modeling conservation of the BCL-2 hydrophobic binding groove

**Fig 3:**
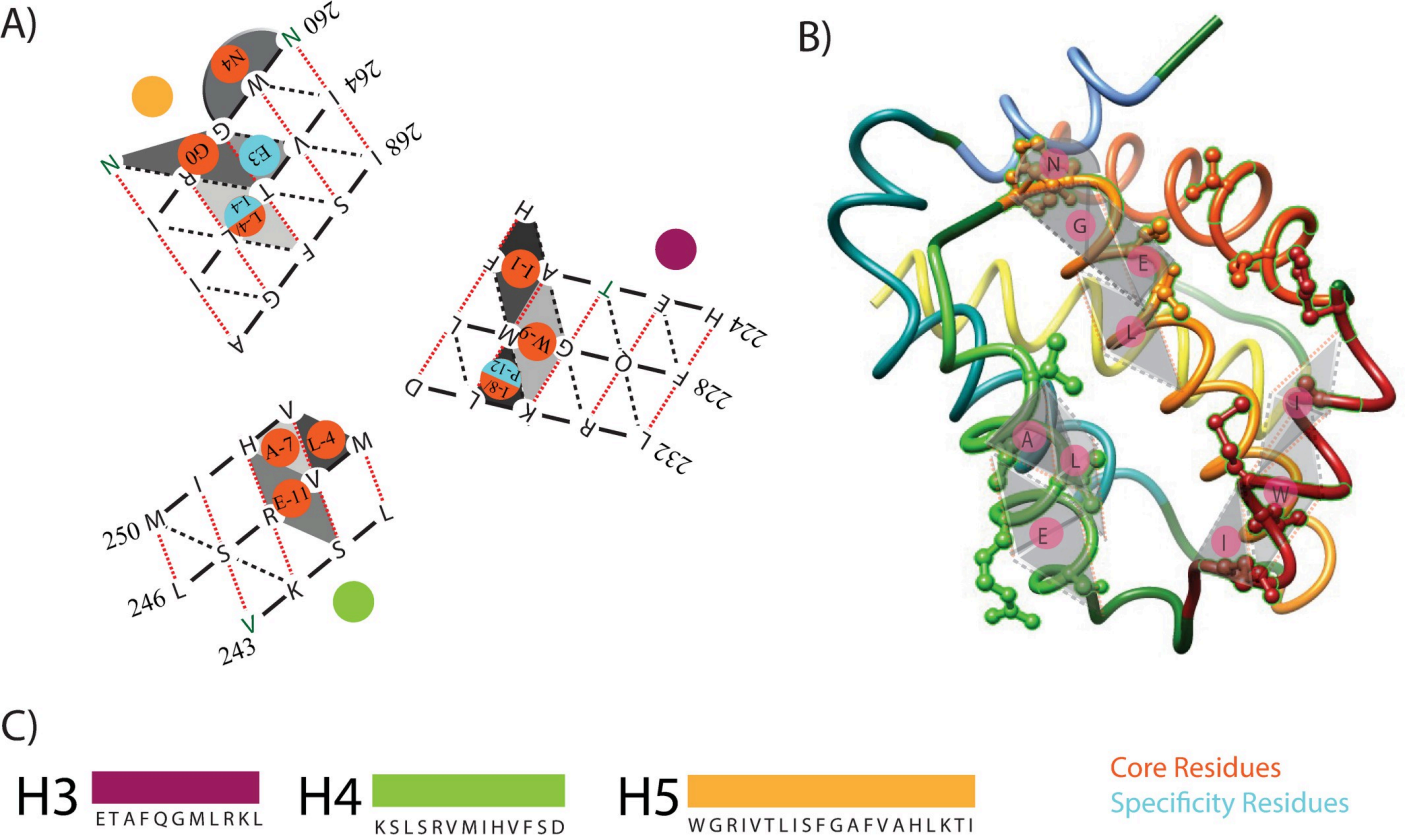
Model of Mcl-1 general binding. Only H3, H4, & H5 are shown. The orientation of the α-helices are rotated to match the binding groove and provide a relative idea of the packing cleft’s surface. (A) The knob-socket diagram of Mcl-1, with only the essential parts of the helices included. The orange knobs represent the residues that are conserved across different pro-survival BCL-3 proteins and could be considered important for binding affinity. The blue knobs represent the residues that specific to the pro-survival BCL-3 protein homolog, and most likely contribute to binding specificity. (B) 3D model of Mcl-1 generated from UCSF Chimera (16). The important sockets and knobs have been overlaid. (C) The amino acid sequence of Mcl-1, organized based on the helices H3, H4, and H5.

As an example, Fig 3 shows the binding groove of the BCL-2 protein Mcl-1 and identifies the essential knob residues from BH3 ligands that are conserved for Mcl-1 binding. Interestingly, the binding groove surface consists of only socket contributions from only 3 α-helices: H3, H4, and H5. The conserved residues that are present in the same position in all Mcl-1 binding interactions with various BH3 peptide complexes constitute the core of the Mcl-1 protein and are shown in red in Fig 3. All other residues that participate in the binding interaction but are not conserved across all interactions of a certain type imply that these residues are specific to those particular interactions. As the most conserved interaction, every BCL-2:BH3 interaction core is constructed around a central packing of between two glycine residues: one on the BCL-2 protein H5 and the other towards the C-terminus of the BH3 ligand. This central conserved glycine-glycine packing plays a significant role in the binding interactions by properly orienting the ligand in the hydrophobic binding groove. The BH3 helix ligand uniquely packs itself in the BCL-2 hydrophobic groove based on the central Gly residue that has close packing with a Gly from H5 of the binding groove. Essentially, these glycine residues provide a point of reference for the remaining binding interactions. Furthermore, the BH3 ligand Gly knob packs into two contiguous sockets or a pocket on Mcl-1 formed by an N-terminal H5 helical socket and a hybrid socket of a coil residue with 2 H5 helical residues. Using the BH3 ligand glycine as a reference point or Gly0 relative to the other residues, the remaining conserved residues on the BH3 α-helix packing into H5 of Mcl-1 are Asn4 and Leu-4, residues 4 after and 4 before the reference glycine, respectively. The Asn4 packs a socket of three consecutive residues at the N-terminus of H5. The Leu-4 packs into the diamond α-helix pocket of R:TL:F, where a pocket is 2 contiguous sockets packing the same knob. If there is an Ile at the -4 position and a Met at the 3 position in the BH3 ligand, then α-helix ligand packs changes into BCL-2 instead of MCL-1. The BH3 ligand packing into H5 N-terminus is common across all BCL-2 proteins. For Mcl-1 H4, the packing is all conserved residues Leu-4, Ala-7, and Glu-11 on BH3 ligands. The sockets on McL-1 H4 cluster, where Leu-4 packs into the VM:V socket, the Ala-7 packs into the V:HV socket, and the Glu-11 packs into the S:RV:H pocket. For McL-1 H3, the packing is similar to the classic coiled-coil between 2 α-helices with the conserved residues Ile-9 packing into the H:KL socket, Trp-8 packing into the A:GM:K pocket, and the Ile-1 packing into the H:AF:M pocket. A hallmark of BIM BH3 domain binding is a Pro-12 that packs into the H:KL socket on MCL-1, which demonstrates that ligands have unique binding modes to the same protein.

### Consensus modeling of BH3 α-helix ligands

The Knob-Socket packing topology maps for BH3 ligands with respective BCL-2 knobs were analyzed for conservation. A full set of maps are provided in S1 Fig. From the 19 Knob-Socket packing topology maps, a model for the BCL-2 protein binding cleft and for the BH3 ligand is developed and presented based on the socket surfaces and their corresponding knobs that pack into those sockets. Orientation of the BH3 ligands was referenced against the central Gly residue that packs into the BCL-2 H5. These knob-socket interaction maps of BH3 ligands with their respective BCL-2 protein knobs were considered. Essentially, the knob residues of the BCL-2 helices are projected onto the sockets of the BH3 α-helical lattice. Knobs and sockets that are conserved based on each BCL-2 partner. In this way, the diversity of knob-socket residues and binding patterns across different BCL-2:BH3 combinations could be identified.

## Results and discussion

### Packing topology of BCL-2:BH3 binding

**Fig 4:**
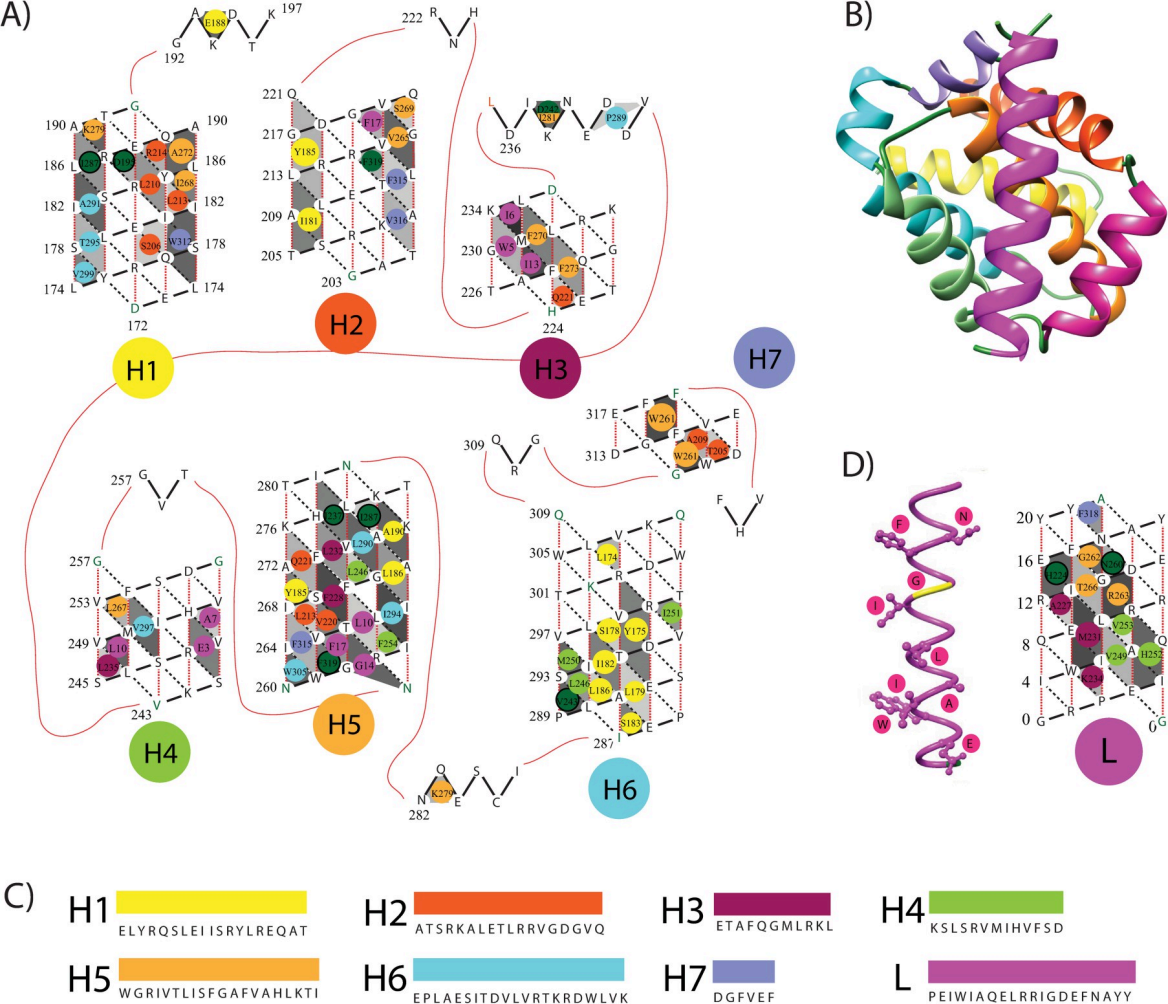
BCL-BH3 binding. Full map showing the interaction between Mcl-1 and Bim. (A) The 2D knob-socket representation of the protein, with the knobs color-coded to identify the helix of origin. (B) A 3D model of the interaction generated by UCSF Chimera (16). (C) The amino acid sequences of all of the helices with corresponding color coding. (D) Side by side, 3D coil representation and 2D helix lattice models of the ligand. In the 3D model, all of the residues that serve as knobs have been shown and labeled.

Fig 4 shows a representative from the 19 Knob-Socket packing topology maps of the BCL-2:BH3 interactions (listed in Table 1). Clearly, the BH3 domain α-helix binds into a groove mainly formed by BCL-2 sockets on helices H3, H4, and H5 with a single socket from helix H2. While predominantly hydrophobic, this groove also consists of charge, hydrogen bonding, and a unique glycine-glycine packing interaction. Each of the H3, H4, and H5 surfaces packs against a trio of residues from BH3. The BCL-2 H3 sockets form surfaces in a classic coiled-coil packing motif with aliphatic Ile and a bulky aromatic Trp from the BH3 α-helix. The BCL-2 H4 sockets pack against the BH3 ligands charged Glu and aliphatic residues Leu and Ala. The BCL-2 H5 sockets pack into an aromatic Phe, aliphatic Leu, and an important Gly, that is highly conserved across BH3 ligands. The BCL-2 H2 socket V:GV interacts with a single aromatic Phe group from the BH3 ligand. Conversely, the amphipathic BH3 ligand presents a set of sockets that bind knob residues from the BCL-2 H3, H4, and H5 helices as well as a single residue from H7 and two residues from coil regions C2-3 and C4-5. Surprisingly, there are more charged/polar residues binding than hydrophobic. The BCL-3 H3 knob residues Ala, Met, and Lys pack in a classic coiled-coil orientation with the BH3 domain’s sockets. BCL-3 H4 and H5 also contribute clusters of knob residues that pack into sockets on the BH3 ligand. The BCL-3 H4 packs 2 aliphatic Val and a charged His and BCL-3 H5 packs the conserved Gly, polar Thr, and charged Arg.

The residues that participate in the knob-socket binding interactions between the pro-survival BCL-2 proteins and BH3 domain complexes can be classified as either conserved residues or variable residues. Conserved residues refer to residues that are conserved across all BCL-2 and BH3 interactions. These conserved residues represent the minimum set of residues needed in order to bind the other protein. Variable residues are those that differ amongst various BCL-2 and BH3 interactions and are unique to every binding interaction. The conserved residues across all of BH3 ligands could be thought of contributing to the general affinity of BCL-2 groove binding, whereas the variation can be thought of as increase binding specificity and provide extra stability for these interactions. Residues that do not participate in the binding interactions are referred to as non-essential residues. By providing these models, not only is there a clear model of what residues bind, but the surfaces defined by socket residues provide a reason as to why those knob residues are preferred.

### Conservation of the Bcl-2 hydrophobic binding groove

By defining the BCL-1 binding cleft as a set of sockets allows the potential to identify other molecules that could bind as a potential BH3 mimetics. As shown in Table 2, the binding areas on the 3 α-helices all less than 10 Å from each other, although barely. This distance is within the range of small organic molecules. The sockets also provide the chemistry that is preferred in binding at those surfaces. While the Gly packing is important for orientation of BCL-2:BH3 interaction, any of the conserved sockets on the H3, H4, and H5 α-helices are worthwhile targets. The chemistry of binding on the H3 sockets consists of aliphatic methyls in the H:AF:M pocket and M:KL socket as well as aromatic interactions in the A:GM:K pocket. The H4 binding sockets also pack aliphatic methyl groups in the V:HV and VM:V sockets along with carboxyl group in the S:RV:H pocket. The N-terminal packing of H5 involves methyl group packing in the R:TL:F pocket, methylene in the N:GR:T hybrid coil/helix pocket, and amino group packing by the NWG contiguous socket. The distances along with the chemistries can be used to query small molecule databases for potential BH3 mimetics. Potentially, only the 2 or even 1 of the α-helical binding surfaces need to be targeted, since binding at any would inhibit BH3 binding.

**Table 2:** Minimum and maximum distances both within and between helices provided. Coordinates for knob residues were calculated by averaging coordinates of Cβ atoms of participating socket residues. Cα atom coordinates were used if residue was a glycine.

Distances between Knob Residues of Mcl-1 Binding Pocket			
Minimum Distance (Å)			
	H3	H4	H5
H3	4.34	9.41	9.80
H4	–	2.41	7.20
H5	–	–	3.53

Maximum Distance (Å)			
	H3	H4	H5
H3	8.37	14.94	22.39
H4	–	5.24	20.35
H5	–	–	9.94

### Conservation in BH3 α-helix ligands

**Fig 5:**
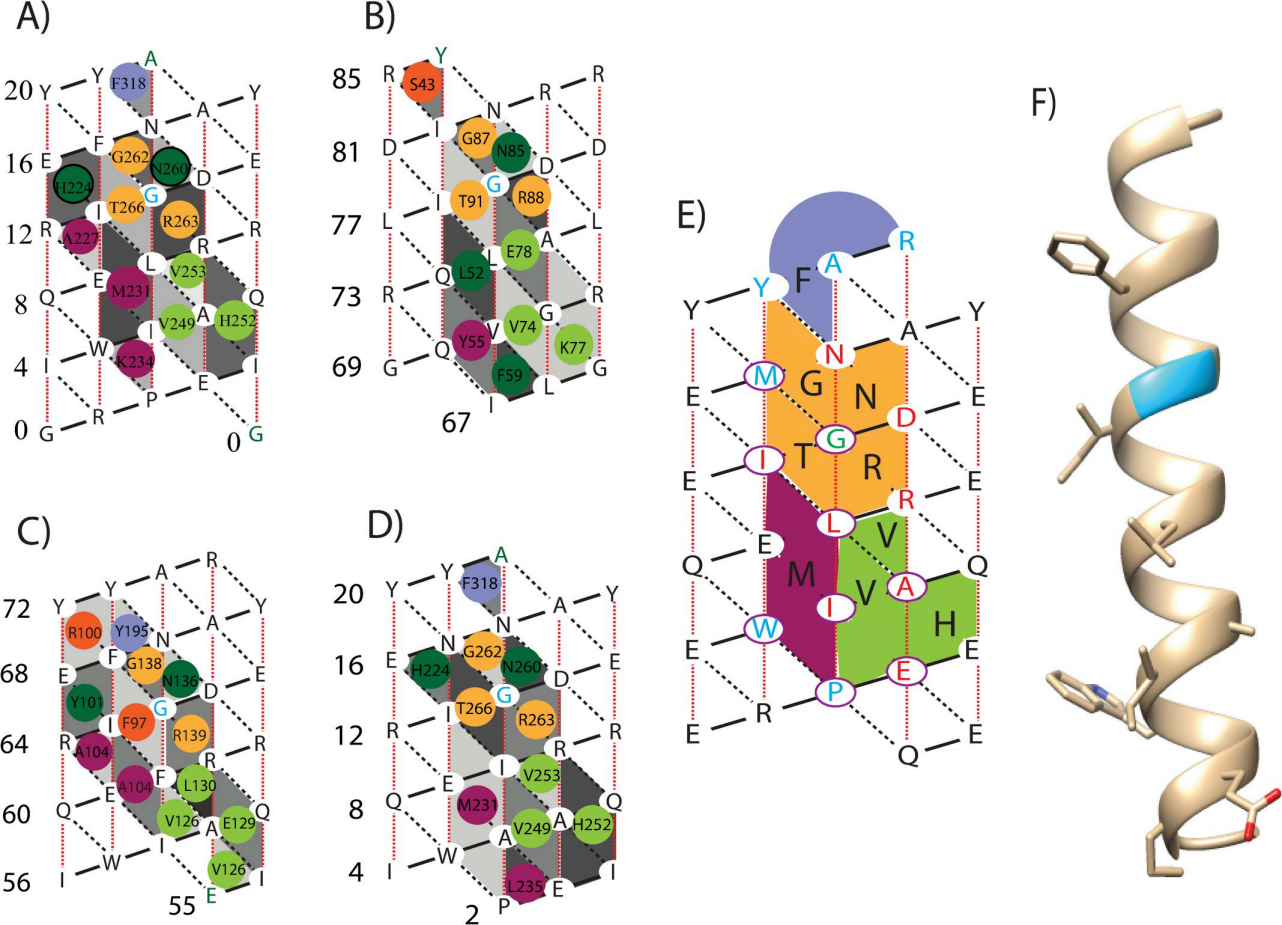
Designed ligand. (A-D) Selection of representative ligand helices from pro-survival protein-BH3 domain complexes. (A) BIM helix in complex with Mcl-1 (PDB: 2pqk). (B) BAK in complex with A1 (2voh). (C) BIM L12F in complex with BCL-XL (3io8). (D) MB7 in complex with Mcl-1 (3kz0). (E) Designed helix with specificity for Mcl-1, showing predicted interactions with protein residues. Conserved residues are shown in red and variable residues that increase specificity for Mcl-1 shown in blue. Central glycine is shown in green. Residues outlined in purple represent knobs that project into the sockets of Mcl-1. (F) 3-dimensional representation of peptide with important interacting residues shown (based on BIM).

Fig 5 shows Knob-Socket packing topology maps for BH3 ligands with respective BCL-2 knobs. Fig 5A–5D are the knob-socket interaction maps of four BH3 ligands with their respective BCL-2 protein knobs. Essentially, the knob residues of the BCL-2 helices are projected onto the sockets of the BH3 α-helical lattice. A full set of maps are provided in S1 Fig. The first three α-helix lattices (Fig 5A–5C) are each to a different BCL-2 partner. These figures demonstrate the diversity of knob-socket residues and binding patterns across different BCL-2:BH3 combinations. Even so, clearly, the conserved and repetitive interactions can be observed, especially the central Gly residue in light blue, which is used to orient the BH3 ligand into the BCL-2 binding site. Also, while packing in the BCL-2 binding sites show variability, the target helices remain the same H3, H4, and H5 with knobs from H7 and coil regions. The variations are mainly in how much interaction with each of the 3 α-helices they interact.

Comparing Fig 5A and 5D, these BH3 ligand α-helices both bind Mcl-1, which can be seen in their similar binding patterns. Fig 5E shows the lattice of a helix designed to mimic a BH3 domain that is specific to Mcl-1. This designed helix represents a consensus model of the BH3 peptide sequence based on interactions between Mcl-1 and various BH3 ligands. The residues of the α-helix lattice are colored according to conservation, where the central Gly is colored green, conserved binding residues are colored red, variable binding residues are colored blue, and non-binding solvent exposed residues are colored in black. Those residues circled in purple represent knob residues of the consensus helix that are predicted to interact with sockets on Mcl-1. The colored sockets represent the predicted knob residues of the Mcl-1 helices that will interact with the designed helix. and all other positions in the BH3 helix ligand can be labeled in relation to this glycine. As shown in Fig 5E and 5F, most of the binding interactions occur around and below the central Gly residue.

There are 3 residues conserved across the BH3 ligands: a central packing Gly, a neighboring Asp and Leu. The central Gly is one of the most conserved residues, as it is present in nearly every BCL-2:BH3 interaction. Out of the 19 interactions studied, there were three interactions that did not contain a central Gly: murine BFL-1:BMF, murine BFL-1:PUMA, and BCL-xL:BAD. In the case of the complexes involving the BCL-2 protein murine BFL-1, the BH3 peptide lacks a central Gly and instead has an Ala in place of it. This implies that the central glycine residue may be substituted with other residues that accomplish the same role across different species. In the BCL-xL:BAD interaction, the residue that occupies the usual position of central Gly is a Ser. Because of this conservation, the central Gly can serve as a reference point in each BCL-2:BH3 interaction, and in most BH3 ligand peptides, the central Gly occupies position 20. The most conserved residue in these BCL-2:BH3 interactions is the Asp residue that occupies the +1 position 21. Unlike the central Gly, this Asp residue is present in every interaction listed and mapped in Table 1 in the 21st position directly adjacent to the central residue. Interestingly, it is not involved in binding as a knob, but rather must form a very important socket surface. Other highly conserved residues, though less so than the central Gly, include Ile in the 19 position, Leu in the 16 position, and Asn in the 24 position. Generally, it appears as though the residues closest in proximity to the central glycine core are more conserved than those further in proximity from the central Gly.

**Fig 6:**
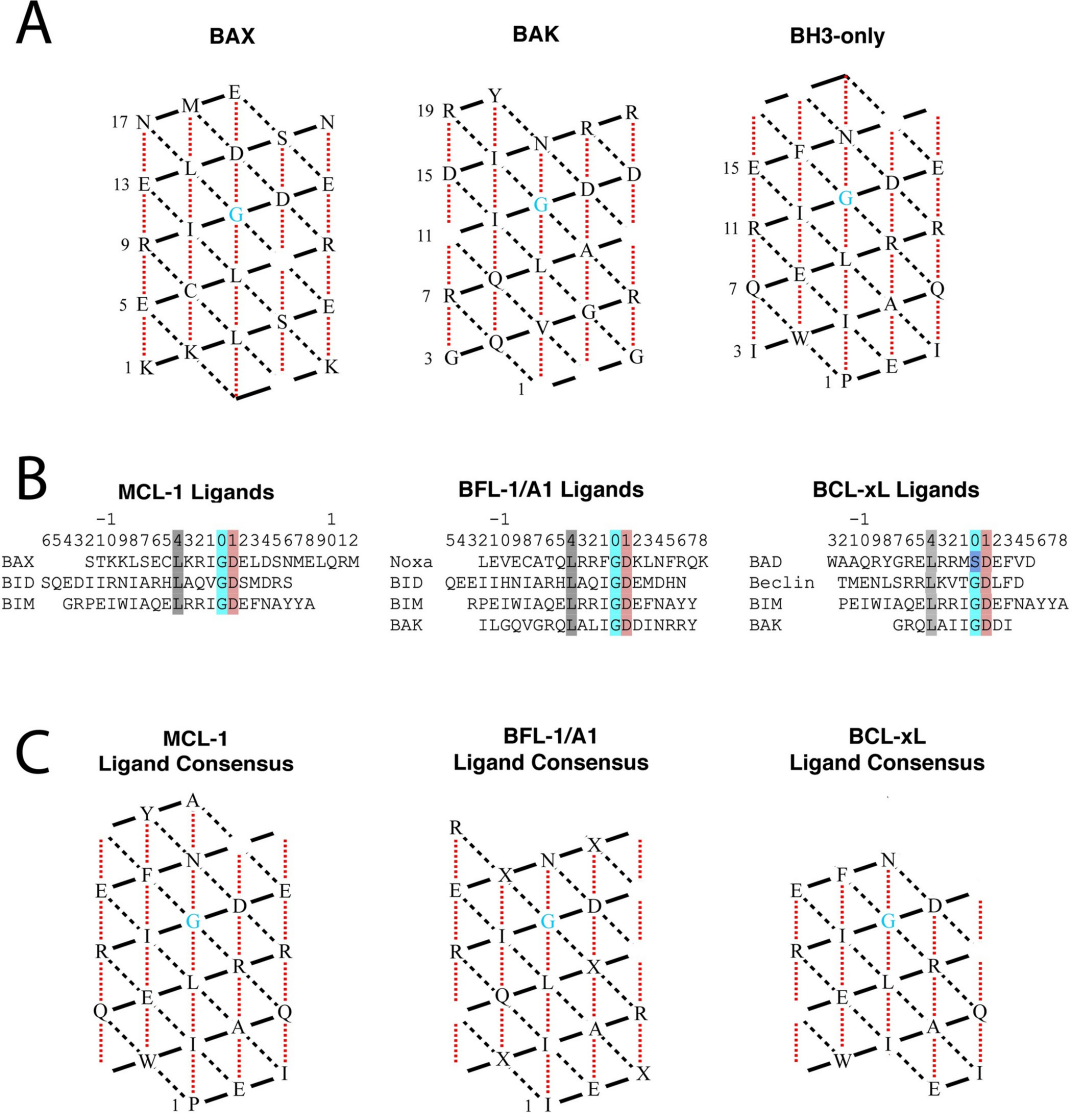
BH3 domain consensus models. A) Consensus models of BAX, BAK, and BH3-only proteins. The essential residues of the BH3 domain for each type of protein are represented on a 2-dimensional lattice of the α-helix. The central glycine is indicated in blue. Blank residues represent non-essential residues that do not participate in the binding interaction between the BH3 peptides and the BCL-2 protein paralog. Residues denoted by ‘X’ represent residues where the consensus residue could not be determined (due to lack of consensus in the chosen set of BH3 peptides). B) Sequence alignments for the unique BH4 ligands of Mcl-1, BFL-1/A1, and BCL-xL are shown. Sequences of point mutants were omitted for clarity. The conserved Gly, Asp, and Leu are highlighted. C) From these sequence alignments as was done in part A, consensus models for BH3 peptides that bind Mcl-1, BFL-1/A1, and BCL-xL. Each 2-dimensional lattice on the bottom row represents the consensus sequence of the BH3 peptides that bind to the respective BCL-2 protein paralog listed in Table 1. The residues shown are those that appeared the most frequently for that BH3 ligand when bound to various pro-survival BCL-2 proteins. Note that the consensus sequence of the BH3 domain ligands is not necessarily the most optimal sequence for binding the BCL-2 protein. These models are only representative of the selected BH3 peptides (Table 1). The number and types of BH3 helices used in the consensus sequence varies for each BCL-2 protein paralog.

The Bax, Bak, and BH3-only proteins are members of the BCL-2 protein family that each contain a BH3 domain that binds into the cleft of the BCL-2 protein. As in Fig 5E, consensus models of Bax, Bak, and BH3-only α-helix ligands were created to compare the sequences of the BH3 ligands that interact with the BCL-2 cleft (Fig 6A). These consensus models indicate the essential residues of the BH3 domain shared amongst a variety of BH3 ligands when interacting with BCL-2 proteins. Each consensus model highlights the central glycine of the BH3 ligand along with the surrounding essential residues that indicate knob-socket interactions with the various BCL-2 proteins. The similarities across all three models involve the central glycine as well as the residues closest in proximity to the glycine that interact with H5 of BCL-2. However, these models also show the diverse sequence variability between the BH3 domains of Bax, Bak, and BH3-only proteins. While the solvent exposed residues (shown on the edges of the lattices) are expected to vary, the residue variability in packing occurs in the interactions with H3 and H4. In particular, at -7 from the central Gly, the position is Ser, Gly, or Ala that pack into the V:HV socket of H3, and at -8 from the central Gly, the position is an L or V or I, which all packing in the M:KL socket of H4. In Fig 6B are sequence alignments and Fig 6C the consensus sequences of the BH3 domain helices that bind into particular BCL-2 proteins. Again, conservation is seen at and around the central Gly with interactions between the BH3 ligand and the BCL-3 protein H5, which may indicate these are primarily stabilizing interactions. The variability between the BH3 domain sequences occur with knobs and sockets involving the BCL-3 H3 and H4, which imply these residues are involved in the differential recognition of BCL-3 protein homologs.

To further investigate the conserved residues forming the core of binding and the variable residues allowing for specificity, the binding was split into a table according to knob residue interaction with corresponding sockets (S6 Fig). Across all studied complexes, there are several binding patterns that are conserved in every BCL-2:BH3 interaction. S6 Fig shows the conserved residues in all BCL-2:BH3 interactions modeled after the Mcl-1 protein interaction and depicted as knob residues from the BH3 peptide that project into the sockets of the BCL-2 protein. The lattice on the top left of S6 Fig represents the 27 numbered positions of the BH3 helix. The circled numbers indicate the conserved knob residues that bind into the BCL-2 sockets. In the table of S6 Fig, the images in the right column show the varying socket residues and positions into which the conserved knobs bind. The identities of these knob residues differ across BCL-2:BH3 combinations; the possible residues are listed in parentheses below the corresponding binding pattern. The four conserved knob residues Glu9, Ala13, Gly20 and Leu23 of the BH3 peptide can be seen binding into the sockets of the H3 and H4 helices. These four residues are conserved across all BCL-2:BH3 interactions investigated in this study. Similarly, there are certain knob residues from the BCL-2 protein that consistently project into the sockets of the BH3 helix (S2 and S6 Figs). Although the exact positioning and socket residues may vary across different BCL-2:BH3 combinations, the most common binding pattern conserved across all interactions (in Table 1) is depicted (S2 Fig).

Variable residues are those that vary across the BCL-2 protein paralogs, but are conserved within a particular BCL-2 protein. These residues contribute to binding specificity and constitute binding patterns that are specific to that BCL-2 protein. S6 Fig shows the variable binding patterns that differ across BCL-2 protein paralogs, with the knobs of the BH3 helix projecting into the BCL-2 sockets. The depicted residues are those that are specific to certain BCL-2 protein paralogs. Even if a BH3 residue is conserved, there are still many slight variations as to how that knob residue will bind into the sockets of the BCL-2 protein and occur at residues 8, 11, 12, 15, 16 and 19. These variable residues also consist of BCL-2 knobs binding into the sockets of the BH3 helix. In the example of Mcl-1, all six interactions involving the Mcl-1 protein contained the following Mcl-1 knob residues: Val, His, Phe, and Met. (S3 and S6 Figs). These binding patterns help inform the sequence of the BH3 residues and depict the predicted interactions of the BH3 helix with the BCL-2 protein.

## Conclusion

In this study, the packing of the binding interfaces between 19 BCL-2 proteins and BH3 ligand α-helices were mapped using the Knob-Socket analysis. The goal is to identify common binding patterns amongst various iterations of the BCL-2:BH3 complex in terms of residue knobs packing particular surfaces defined by three residue sockets. Through this research, multiple conserved residues and binding surfaces were identified across all studied BCL-2:BH3 interactions, as well as those specific to certain BCL-2 protein paralogs. Decreasing the concentration of active BCL-2 in the cell by saturating free pro-survival BCL-2 proteins with peptides that mimic the BH3 domain will prevent BCL-2 from inhibiting the process of apoptosis, allowing cancerous cells to die. This research presents an outline for the rational design of potential peptides small molecules that mimic the BH3 domain and bind into the BCL-2 protein binding pocket. The results of this Knob-Socket analysis can hopefully provide more focus in the screening libraries of peptides and small molecules as cancer therapeutics.

## Supporting information

S1 Fig Knob-Socket maps of ligand BCL-2:BH3 interactions. Visual representations of the interactions listed in Table 1. The knobs of the BCL-2 proteins are shown packing into the sockets of the BH3 helix ligands.
(TIFF)

S2 Fig Conserved binding patterns of BCL-2 Knobs packing into BH3 sockets. The PDB IDs of each BCL-2:BH3 interaction is listed next to the binding pattern variation it corresponds to. Less common residues are shown in parentheses. Two-dimensional lattice of the BH3 helix is shown in top left.
(TIFF)

S3 Fig Variable binding patterns of BCL-2 Knobs packing into BH3 sockets. The PDB IDs of each BCL-2:BH3 interaction is listed next to the binding pattern variation it corresponds to. Less common residues are shown in parentheses. Two-dimensional lattice of BH3 helix is shown in top left.
(TIFF)

S4 Fig Conserved binding patterns of BH3 Knobs packing into BCL-2 sockets. The PDB IDs of each BCL-2:BH3 interaction is listed next to the binding pattern variation it corresponds to. Less common residues are shown in parentheses. Two-dimensional lattice of BH3 helix is shown in top left, with knob residues that pack into BCL-2 protein outlined in purple.
(TIFF)

S5 Fig Variable binding patterns of BH3 Knobs packing into BCL-2 sockets. The PDB IDs of each BCL-2:BH3 interaction is listed next to the binding pattern variation it corresponds to. Less common residues are shown in parentheses. Two-dimensional lattice of BH3 helix is shown in top left, with knob residues that pack into BCL-2 protein outlined in purple.
(TIFF)

S6 Fig Table of conserved residues of the BH3 helix and BCL-2 pocket interaction. For each highlighted conserved residue, indicated is the pocket(s) in which it binds into on the Bcl-2 protein. The two-dimensional lattice of BH3 ligand is shown, with circled knob residues outlined in the color of BCL-2 helix it binds into. Model of the MCL-1/BIM knob-socket map is shown for reference. The colored circles next to each BCL-2 pocket indicates the helix of the BCL-2 protein on which it is located. All possible identities of knob residues are listed in parentheses next to pockets.
(PDF)

S7 Fig Table of variable residues of the BH3 helix and BCL-2 pocket interaction. For each highlighted variable residue, indicated is which Bcl-2 protein it is specific to, as well as the pocket(s) in which it binds into on the BCL-2 protein. The two-dimensional lattice of BH3 ligand is shown, with circled knob residues outlined in the color of BCL-2 helix it binds into. Note that variable residues 16 and 19 each bind into two different helices (each outlined in two different colors). Model of the MCL-1/BIM knob-socket map is shown for reference. The colored circles next to each BCL-2 pocket indicates the helix of the BCL-2 protein on which it is located. All possible identities of knob residues are listed in parentheses next to pockets (residue identity indicated in table for the ‘Surrounding Methionine 231’ residue).
(PDF)

S1 File Data set of structure and KS analysis. (TGZ)
