# Supplementary figures and images for: Characterizing the consensus residue specificity and surface of BCL-2 binding to BH3 ligands using the Knob-Socket model
Source: PLoS One. 2023 Feb 16;18(2):e0281463. doi: 10.1371/journal.pone.0281463 (PMC9934389; doi:10.1371/journal.pone.0281463)

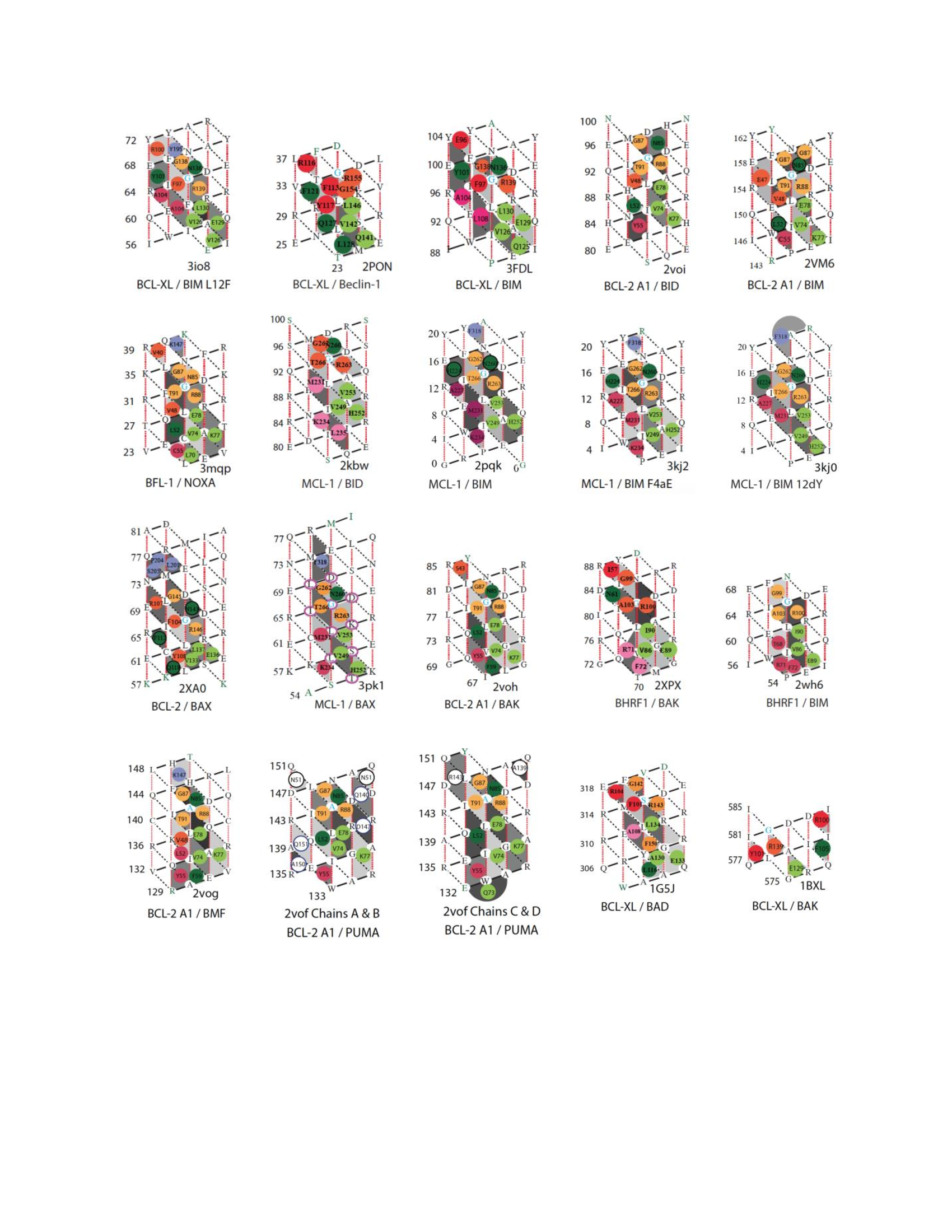

Supplement: S1 Fig — Visual representations of the interactions listed in Table 1. The knobs of the BCL-2 proteins are shown packing into the sockets of the BH3 helix ligands. (TIFF) [file pone.0281463.s001.tiff]

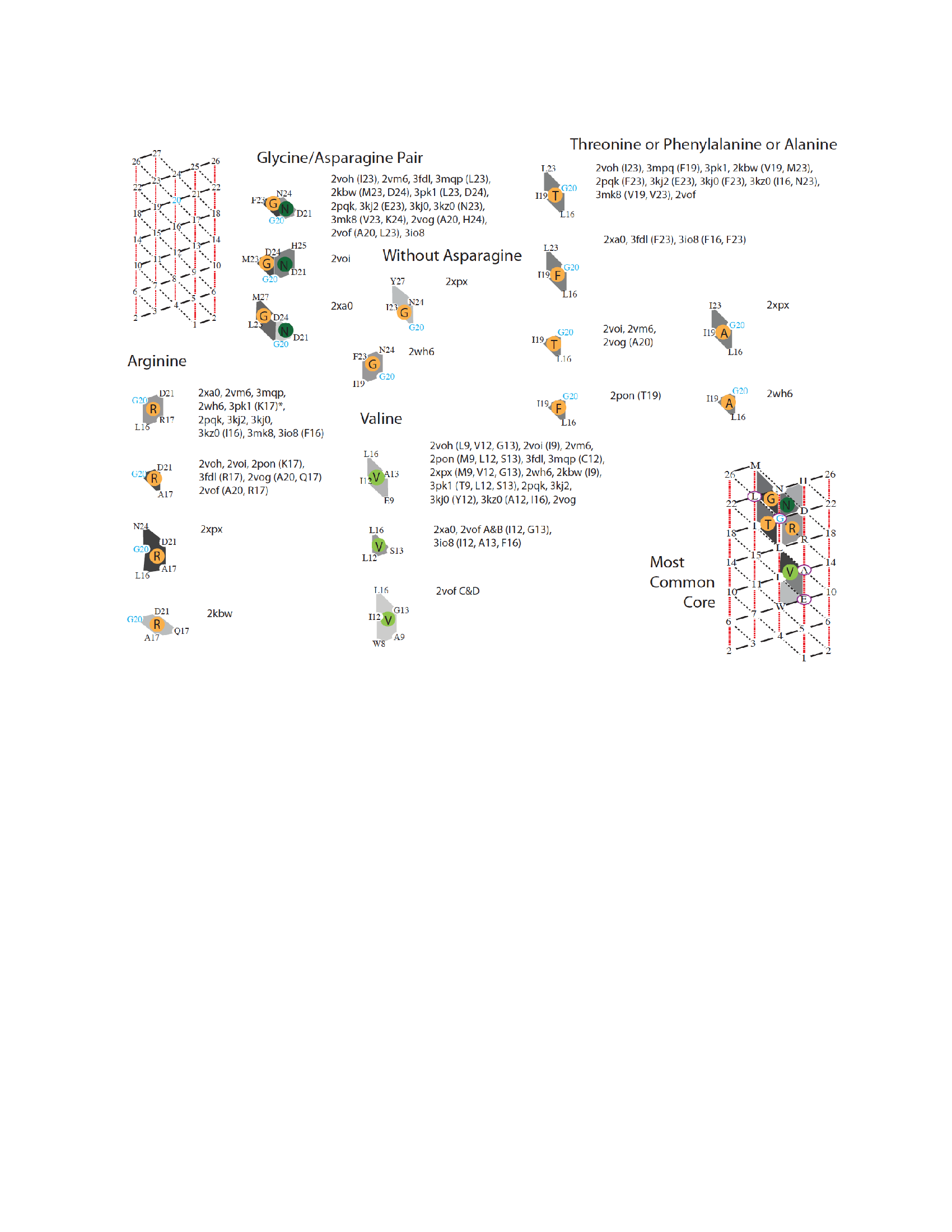

Supplement: S2 Fig — The PDB IDs of each BCL-2:BH3 interaction is listed next to the binding pattern variation it corresponds to. Less common residues are shown in parentheses. Two-dimensional lattice of the BH3 helix is shown in top left. (TIFF) [file pone.0281463.s002.tiff]

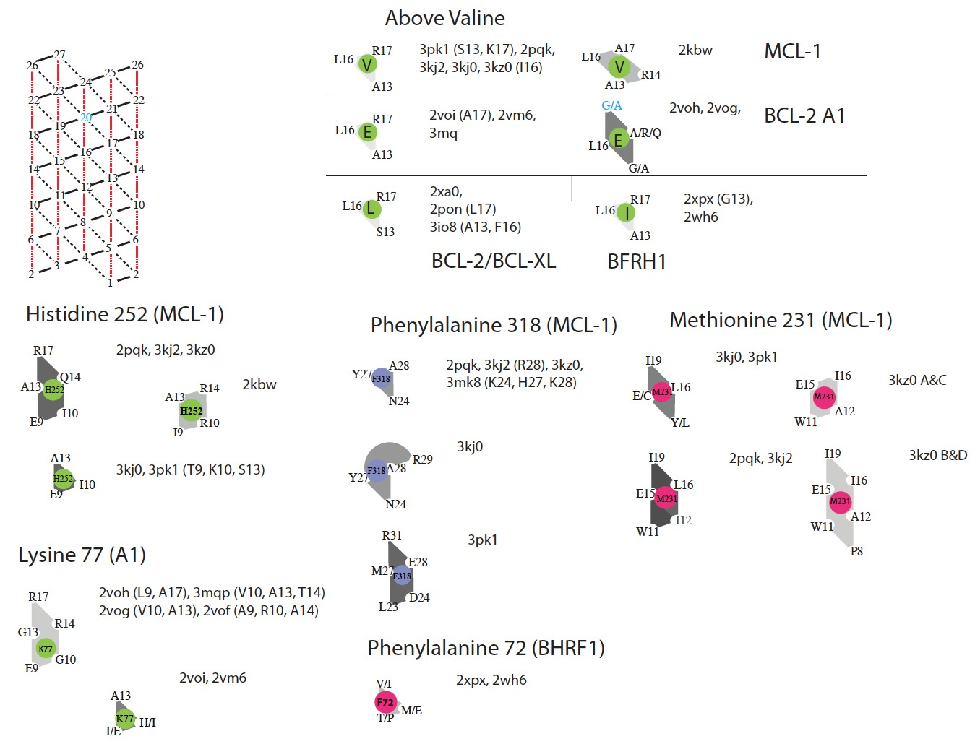

Supplement: S3 Fig — The PDB IDs of each BCL-2:BH3 interaction is listed next to the binding pattern variation it corresponds to. Less common residues are shown in parentheses. Two-dimensional lattice of BH3 helix is shown in top left. (TIFF) [file pone.0281463.s003.tiff]

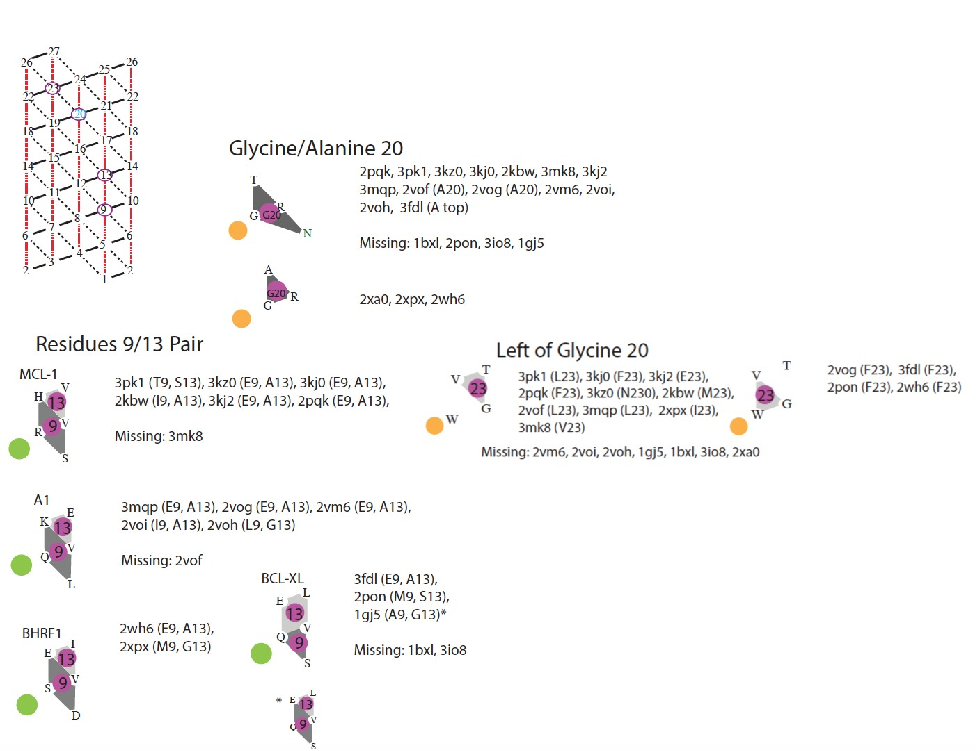

Supplement: S4 Fig — The PDB IDs of each BCL-2:BH3 interaction is listed next to the binding pattern variation it corresponds to. Less common residues are shown in parentheses. Two-dimensional lattice of BH3 helix is shown in top left, with knob residues that pack into BCL-2 protein outlined in purple. (TIFF) [file pone.0281463.s004.tiff]

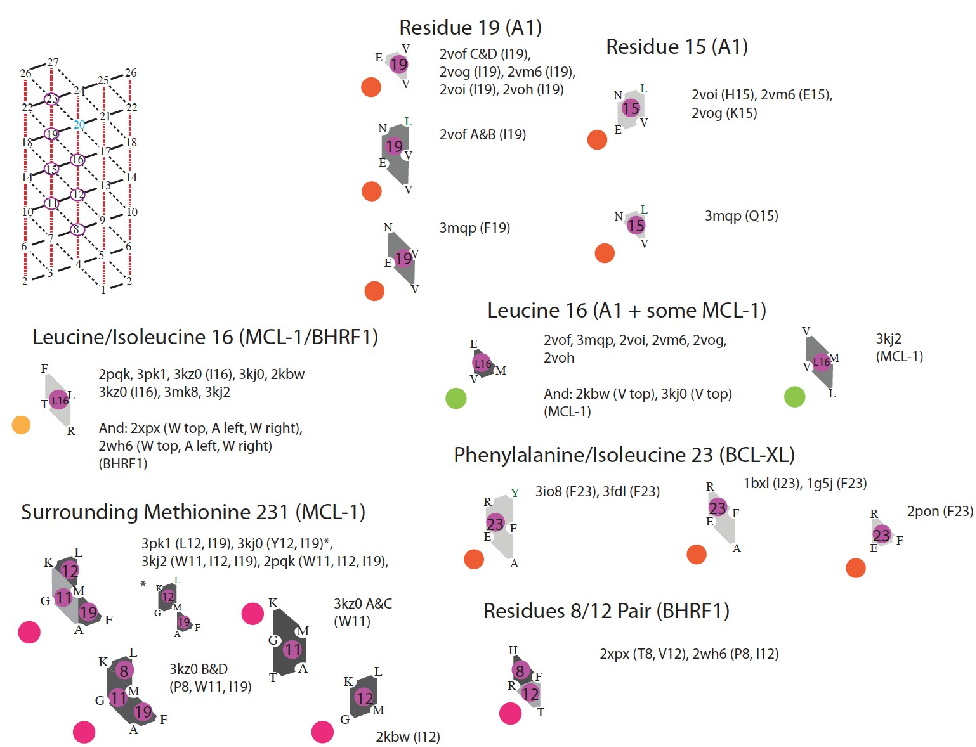

Supplement: S5 Fig — The PDB IDs of each BCL-2:BH3 interaction is listed next to the binding pattern variation it corresponds to. Less common residues are shown in parentheses. Two-dimensional lattice of BH3 helix is shown in top left, with knob residues that pack into BCL-2 protein outlined in purple. (TIFF) [file pone.0281463.s005.tiff]
