# Supplementary material for: Characterizing the consensus residue specificity and surface of BCL-2 binding to BH3 ligands using the Knob-Socket model
Source: PLoS One. 2023 Feb 16;18(2):e0281463. doi: 10.1371/journal.pone.0281463 (PMC9934389; doi:10.1371/journal.pone.0281463)
Supplement: S6 Fig — For each highlighted conserved residue, indicated is the pocket(s) in which it binds into on the Bcl-2 protein. The two-dimensional lattice of BH3 ligand is shown, with circled knob residues outlined in the color of BCL-2 helix it binds into. Model of the MCL-1/BIM knob-socket map is shown for reference. The colored circles next to each BCL-2 pocket indicates the helix of the BCL-2 protein on which it is located. All possible identities of knob residues are listed in parentheses next to pockets. (PDF) [file pone.0281463.s006.pdf]

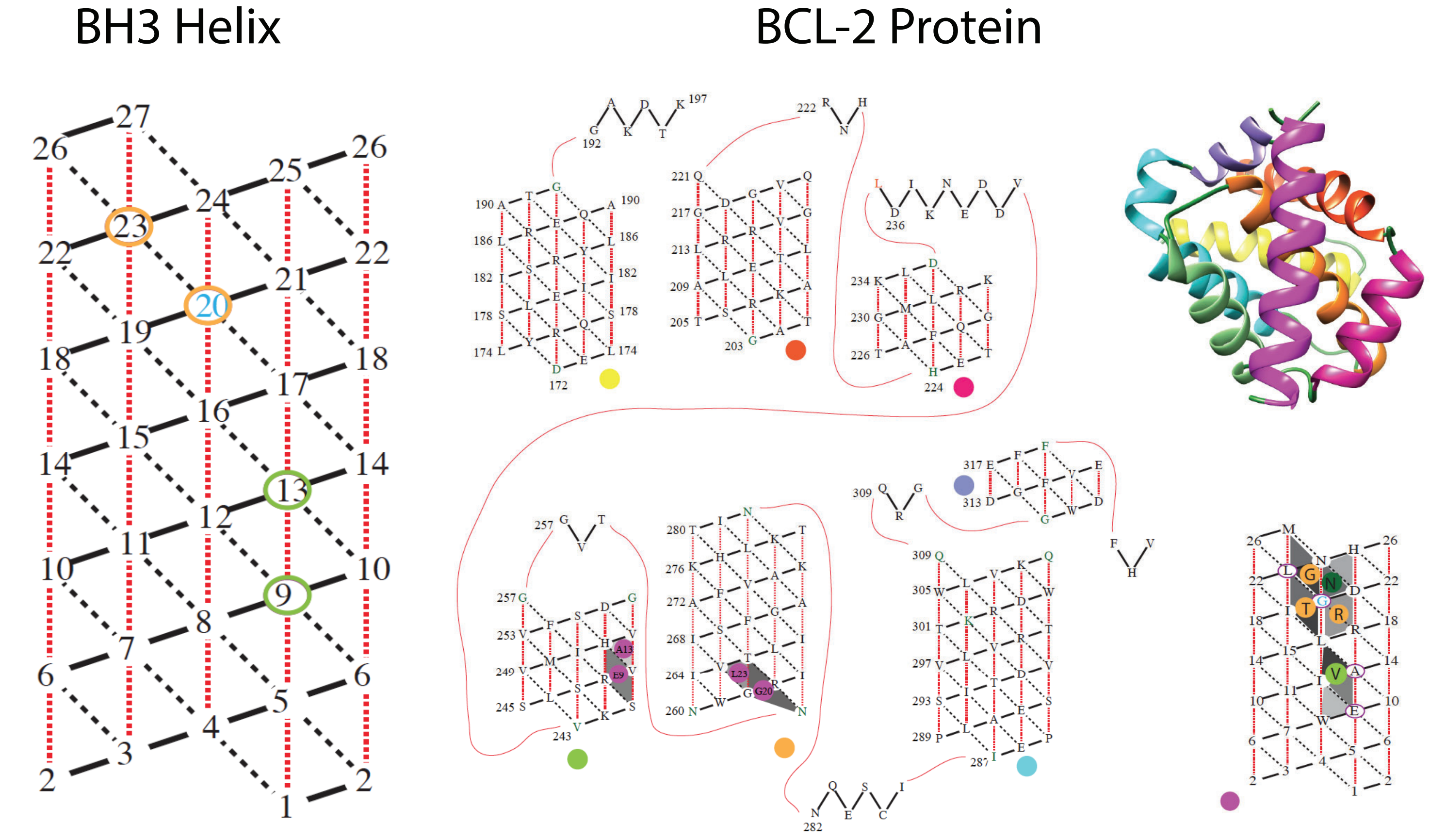

Conserved Residues:

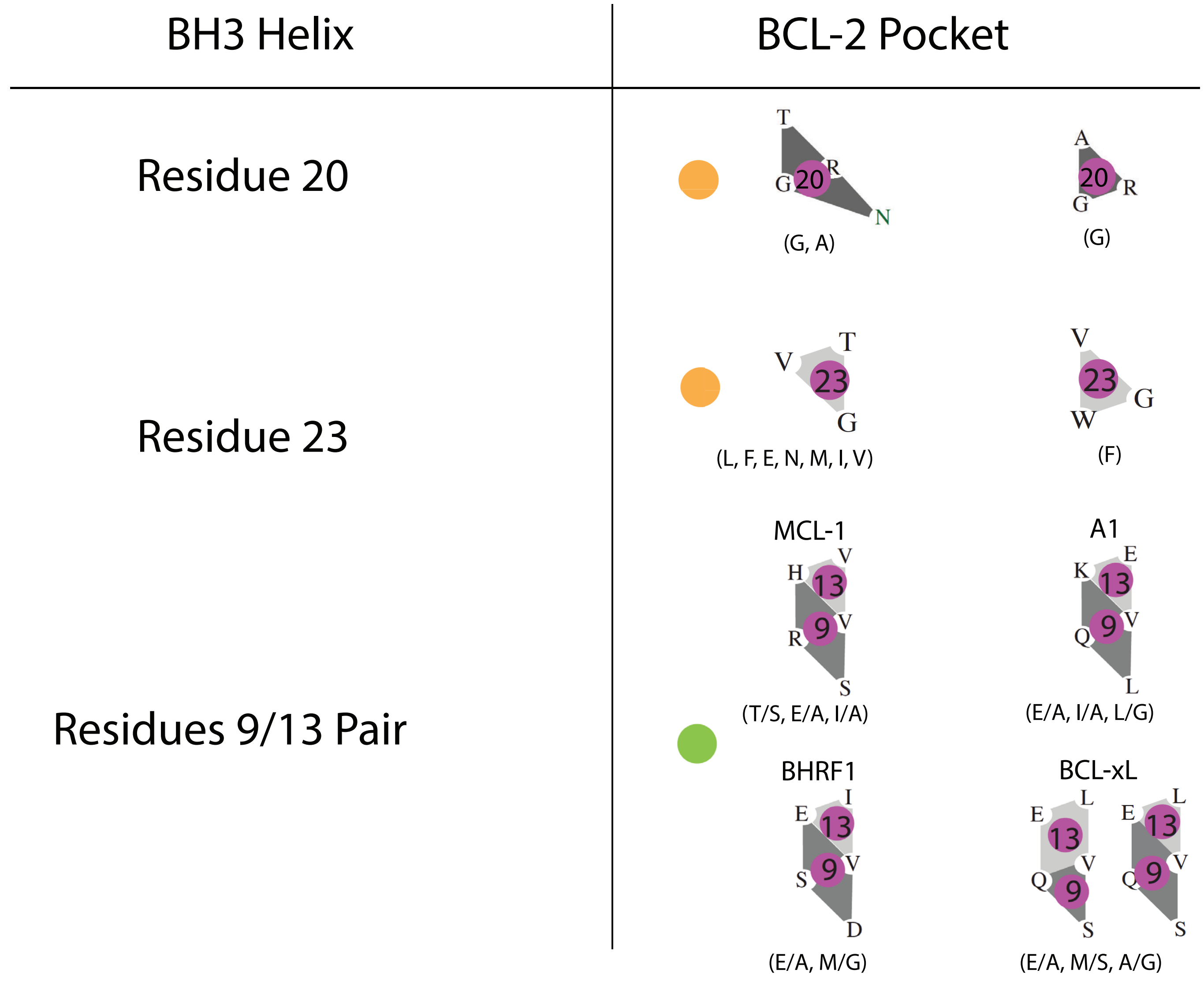

**Colored circle next to each socket indicates the helix of the BCL-2 protein on which it is located. Model of 2pqk is shown for reference. Possible residues listed in parentheses next to sockets.
