# Supplementary material for: Characterizing the consensus residue specificity and surface of BCL-2 binding to BH3 ligands using the Knob-Socket model
Source: PLoS One. 2023 Feb 16;18(2):e0281463. doi: 10.1371/journal.pone.0281463 (PMC9934389; doi:10.1371/journal.pone.0281463)
Supplement: S7 Fig — For each highlighted variable residue, indicated is which Bcl-2 protein it is specific to, as well as the pocket(s) in which it binds into on the BCL-2 protein. The two-dimensional lattice of BH3 ligand is shown, with circled knob residues outlined in the color of BCL-2 helix it binds into. Note that variable residues 16 and 19 each bind into two different helices (each outlined in two different colors). Model of the MCL-1/BIM knob-socket map is shown for reference. The colored circles next to each BCL-2 pocket indicates the helix of the BCL-2 protein on which it is located. All possible identities of knob residues are listed in parentheses next to pockets (residue identity indicated in table for the ‘Surrounding Methionine 231’ residue). (PDF) [file pone.0281463.s007.pdf]

BH3 Helix

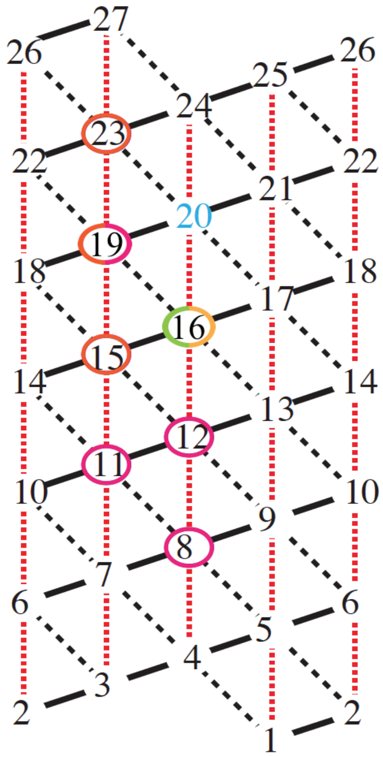

BCL-2 Protein

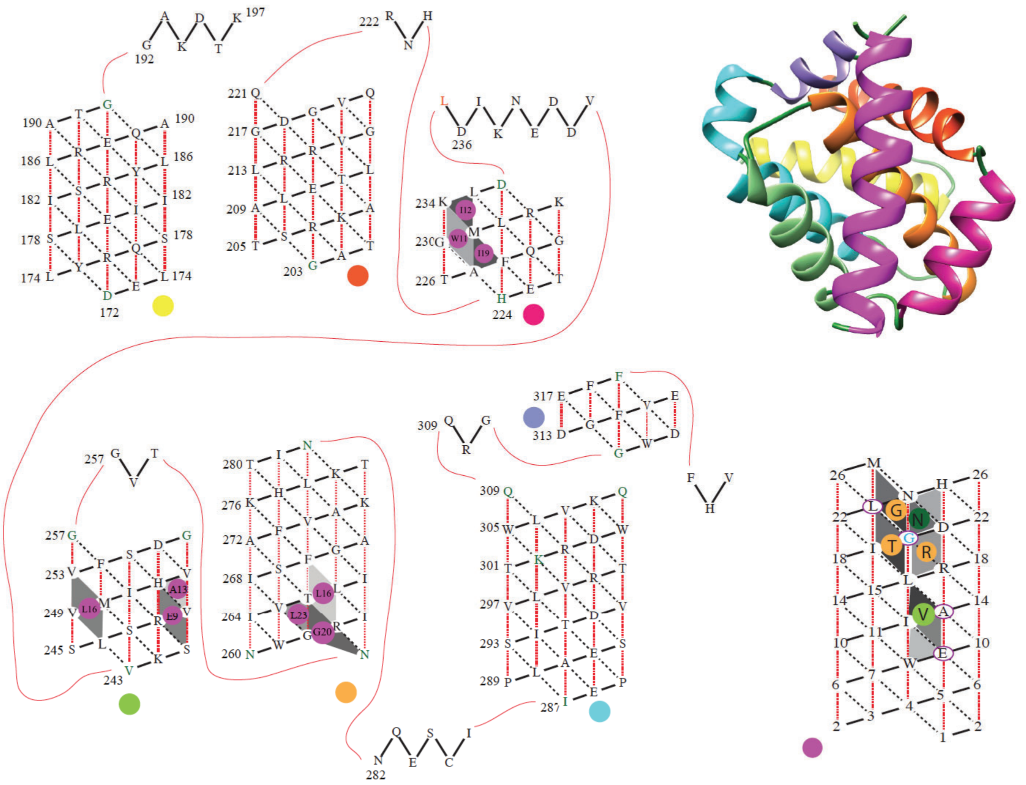

Variable Residues:

BH3 Helix	BCL-2 Protein	BCL-2 Pocket
Residue 15	A1	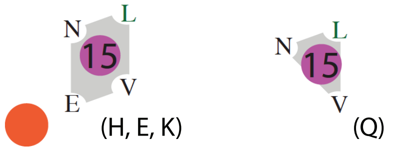
Residue 19	A1	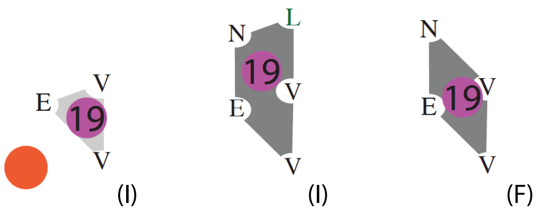
Residue 16	A1 + some MCL-1	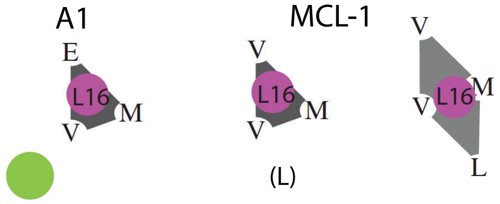
Residue 16	MCL-1 + BHRF1	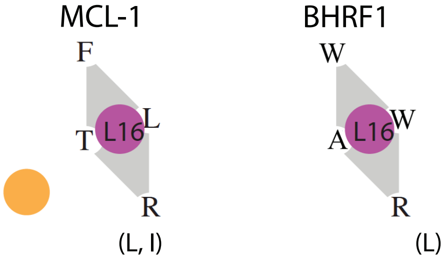

BH3 Helix	BCL-2 Protein	BCL-2 Pocket
Surrounding Methionine 231	MCL-1	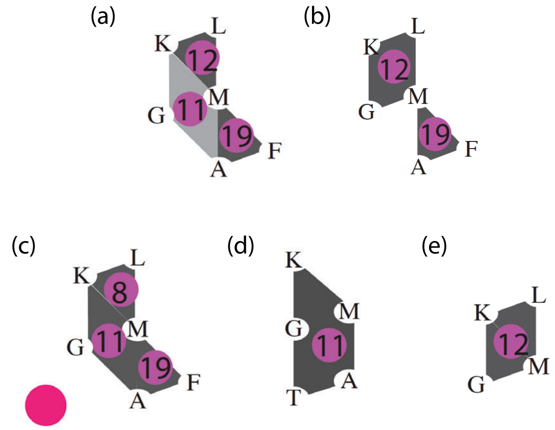
Residues 8/12 Pair	BHRF1	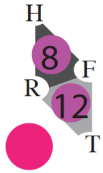 (T/V, P/I)
Residue 23	BCL-xL	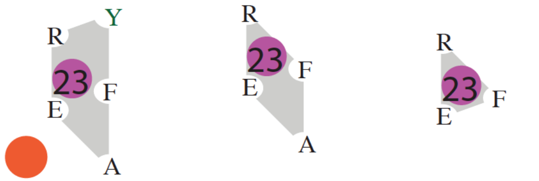 (F) (I, F) (F)

Surrounding Methionine 231:

	a	b	c	d	e
8			P		
11	W		W	W	
12	I	L/Y			I
19	I	I	I		
